# COMPARISON BETWEEN FLEXIBLE NAILING AND EXTERNAL FIXATION, METHODS TO STABILIZE FEMORAL SHAFT FRACTURES IN THE IMMATURE SKELETON: A SYSTEMATIC REVIEW AND META-ANALYSIS

**DOI:** 10.1590/1413-785220243204e278265

**Published:** 2024-10-07

**Authors:** Breno Augusto Giese Ribeiro, Caio Henrique Kenchian, Guilherme Satake, Eiffel Tsuyoshi Dobashi, Amabile Oficiati de Carnevale Galeti

**Affiliations:** 1. Universidade Federal de São Paulo, Esco Paulista de Medicina, São Paulo, SP, Brazil

**Keywords:** Femur Fractures, Child, Fracture Fixation, External Fixators, Intramedullary Fracture Fixation, Systematic Review, Fraturas do Fêmur, Criança, Fixação de Fratura, Fixadores Externos, Fixação Intramedular de Fraturas, Revisão sistemática

## Abstract

Flexible intramedullary nailing and external fixation have become the main methods to surgically treat femur fractures in children. This study aimed to search the current literature and evaluate the clinical and radiographic results of surgical treatment by comparing these methods and investigating their associated complications. This systematic review was carried out following PRISMA (Preferred Reporting Items for Systematic Reviews and Meta-Analysis) recommendations. Searches were carried out on the PubMed, Embase, and Web of Science databases. The search for journals in these databases was carried out from January 2023 to August 2023, retrieving 695 studies. This systematic review included 11 articles, which encompassed 718 patients who underwent surgical external fixation and flexible nailing. The most frequently observed complications referred to late or malunion, superficial and deep infections, skin irritation, angular deformity, and length discrepancy between lower limbs. Both methods of stabilization of pediatric femoral fractures can provide good clinical and radiographic results. However, the choice of treatment with flexible nails is certainly more valid and has greater acceptance than external fixation. ***Level of Evidence III, Systematic Review*** .

## RESUMO

Para o tratamento cirúrgico das fraturas do fêmur em crianças, as hastes intramedulares flexíveis e os fixadores externos tornaram-se os principais métodos utilizados. Este estudo teve como objetivo pesquisar a literatura atual e avaliar os resultados clínicos e radiográficos do tratamento cirúrgico confrontando estes métodos e investigar as complicações associadas. Trata-se de uma revisão sistemática, realizada segundo as recomendações PRISMA (Preferred Reporting Items for Systematic Reviews and Meta-Analysis), cujas buscas foram realizadas nas bases de dados (PubMed, Embase e Web of Science). A busca dos periódicos nessas bases de dados foi realizada entre janeiro de 2023 e agosto de 2023, e foram encontrados 695 estudos. Um total de 11 artigos foram incluídos nesta revisão sistemática, que engloba 718 pacientes que foram operados pelas técnicas cirúrgicas de fixação externa e haste flexível. As complicações mais frequentemente observadas foram a consolidação tardia ou viciosa, infecção superficial e profunda, irritação da pele, deformidade angular e discrepância no comprimento entre os membros inferiores. Verificou-se que ambos os métodos de estabilização das fraturas femorais pediátricas podem proporcionar bons resultados clínicos e radiográficos. No entanto, a escolha do tratamento com hastes flexíveis é certamente mais válida e tem maior aceitação, comparada à fixação externa. **Nível de evidência III, Revisão Sistemática.**

## INTRODUCTION

 The treatment of pediatric femoral shaft fractures (FSF) is based on injury pattern and patients’ age. ^1^

 The American Academy of Orthopaedic Surgeons set guidelines updated in 2015 ^2^ that provide reliable evidence to manage these lesions based on three age subgroups. For fractures in children aged up to five years, it may involve a Pavlik harness, a plaster cast, and/or skeletal traction. At school age, stabilization of these injuries by elastic stable intramedullary nailing (ESIN) constitutes the main choice. However, external fixation (EF) can be used, as in open fractures, multiple fractures, femoral fractures with severe skin lesions, and patients weighing more than 50 kg. 

 Complications from conservative treatment in children aged over five years include reduction loss, vicious consolidation, psychological intolerance (for the child and family), and those associated with the use of plaster casts. Historically, ESIN was introduced to treat femoral fractures by the Nancy group in 1979. ^3^ Titanium is the most commonly used material in these implants due to its excellent biocompatibility and its elasticity, which limits the amount of permanent deformation in the nail during insertion. Its use promotes the formation of stable calluses, limiting stress. ESIN functions as an intramedullary guide that maintains the length and alignment of the fracture, thus enabling rapid mobilization. Such movements biomechanically determine callus formation and may offer a low risk of refracture. ^4^

 Normally, for stable-length fractures, titanium elastic nails (TEN) show high rates of consolidation and require a relatively short period of time before enabling the fractured limb to bear weight. Limited surgical dissection and reduced hospitalization guide the preference for this device. However, complication rates range from 10 to 80% ^47^ , determining reoperations due to length discrepancy between lower limbs, implant migration, malunion, and limitation of use for adolescent patients and those weighing more than 49 kg. ^8^^,^^9^

 Moreover, over the past two decades, ESIN has become a popular choice to fixate femoral shaft fractures in children. ^4^ The technique is based on a three-point support of the nail in the intramedullary canal so the implants occupy at least 80% of its diameter, providing stability and maintaining the reduction without violating children’s growth phases. 

 ESIN treatment show relatively rare complications ^10^ . According to the literature, the most common complication refers to irritation at the protruding ends of the nails, which can cause pain and infect soft tissues and bone. Other complications, such as pseudoarthrosis, malunion, and one-cm limb length discrepancy, occur in 8.2% of preschool children. ^11^

 Therefore, the controversy regarding the efficiency of surgical treatment concerns children aged from five to 11 years due to the variety of therapeutic options and algorithms. Thus, the main strategies for fixation use conventional dynamic compression plates, locking compression plates, limited-contact dynamic-compression plates, submuscular plates, and external fixation. ^5^^,^^10^ The use of plates is indicated, especially for patterns of unstable fractures in length or in children weighing > 49 kg, the benefits of which include decreased incidence of malunion, superior stability in axial and torsional loading, and limited exposure (if the submuscular technique is chosen). ^10^ Comminuted fractures are unstable and thus require surgery even in children. 

 A biomechanical study evaluated pediatric-sized femur models with midshaft transverse fractures that had been stabilized by TEN. It then correlated the results with gait data and suggested that a maximum weight from 40 to 45 kg should serve as the cut-off point for this method of osteosynthesis. Despite such theoretical weight limit, surgeons occasionally use this type of fixation in patients above this weight limit due to the increasing obesity in the pediatric population. ^12^^,^^13^

 External fixation (EF) plays an important role in the treatment of these injuries, especially of unstable shaft fractures. However, several studies have reported significant complications such as pin-track infections, malunion, loss of reduction, and refracture. ^14^^,^^15^

 Only a limited number of studies have focused on the combined use of ESIN and external fixation, such as Erturk et al. ^16^ and Atef and El Tantawy ^17^ , who have used this combination to treat unstable open tibial fractures in adolescents. However, no studies have reported the results of this combination in children aged from five to 11 years with unstable femoral fractures. 

A 2014 Cochrane review evaluated the treatment of FSF in children and adolescents and found no published randomized controlled trials on this topic in the literature. However, several observational comparative studies have been published since the release of the latest American Academy of Orthopaedic Surgeons guidelines.

 We stress the current debate on whether flexible intramedullary nailing or external fixation offer the best surgical method to treat pediatric femoral fractures. ^11^ However, no consensus exist as to which would be the best method to stabilize FSF in the pediatric population. 

Thus, this study aimed to perform a systematic review with a meta-analysis to evaluate the outcomes of flexible nailing versus external fixator to treat femoral fractures in children and investigate the associated complications.

## METHODS

### Type of Study

 This systematic literature review followed the methodological criteria established by Donato & Donato. ^(18)^ This research was carried out according to the Preferred Reporting Items for Systematic reviews and Meta-Analyses (PRISMA) in Galvão et al. ^(19)^

### Research Strategy

 The PICO strategy (P- patient, I- intervention, C- control, O- outcome) was used to establish the search criteria and elaborate the guiding question of this review: “What is the ideal intervention to surgically stabilize femoral fractures in children?” The electronic search was carried out from January 2023 to August 2023 in the following databases: Medical Literature Analysis and Retrievel System Online (Pubmed/Medline) and Science Direct. The search strategy was based on the choice of terms that were obtained from health sciences descriptors. Finally, the references in all retrieved articles were comprehensively examined for other relevant manuscripts. This search was carried out using the following terms: *“Femoral Fractures”* AND *“Child”* AND *“External Fixators”* OR *“Femoral Fractures”* AND *“Child”* AND *“Fracture Fixation, Intramedullary.”*

### Selection Criteria

Primary (cross-sectional, cohort, randomized, and case reports) studies that were conducted on the use of flexible nailing or external fixation in children with femoral fractures and had been published in the last 10 years (without language restrictions) were included.

### Analyzed Data

The titles and abstracts of all retrieved articles were read by two reviewers using a pre-defined search strategy. They applied the inclusion criteria for this review independently. Disagreement was solved by discussion among the evaluators, and an agreement was reached in all cases to establish which studies would be included in this systematic review.

Data were then extracted from the selected studies, including information about their authors, year of publication, study design, participants’ number and characteristics, type of intervention, outcomes, complications, and limitations.

 The Newcastle-Ottawa scale was used to assess the quality of the studies in this review. It evaluates studies by criteria related to selection and comparability between cohorts and criteria related to study outcomes. An adapted list with five aspects of the Newcastle-Ottawa scale was also used to assess the risk of bias based on sample representativeness, exposure, presentation condition, response rate, and result determination. ^20^

### Statistical analysis

 For the statistical analysis, risk ratios and mean differences were estimated with a 95% confidence interval using the random effect model. Heterogeneity was classified based on I ^2^ values: 25%, low heterogeneity; 50%, moderate heterogeneity; and 90%, high heterogeneity. ^21^ All statistical analyses were performed on R, version 4.3.1, using the meta package. 

## RESULTS

### Description of the Search Strategy

 This review screened and evaluated 391 full articles. Assessing their titles and abstracts excluded 374 articles since they failed to meet the chosen eligibility criteria. Selection and analysis rendered 11 articles as eligible to compose this systematic review. This systematic review followed the PRISMA recommendations. The 11 included studies ^12^^-^^14^^,^^22^^-^^29^ evaluated 718 children ( Table 1 ). Observational studies showed moderate quality. 

### Characteristics of the Included Studies

 The included studies were published from 2018 to 2022. They employed retrospective (n = 9) and prospective (n = 2) designs. In total, 437 children underwent internal fixation by ESIN; 234, by EF; and 28, by the combined use of temporary external fixation and flexible intramedullary nailing. Follow-up time ranged from 12 to 24 months. Table 1 shows the epidemiological characteristics of participants. 

**Table 1.** Article Identification

Author/ Year	Type of Study	Sample	Type of Intervention	Follow-up Time
Frumberg et al. ^(^ 22 ^)^	Retrospective Cohort	N: 6 Femur or tibia fracture Sex: 100% boys	Two flexible intramedullary nails	12 months
Li et al. ^(^ 23 ^)^	Retrospective Cohort	N: 71 Fractures of the shaft of the distal third of the femur Sex: 28 girls and 43 boys	External fixation and Flexible intramedullary nailing	24 months
Ulici et al. ^(^ 24 ^)^	Retrospective Study	N: 137 Femoral shaft fractures Sex: 44 girls and 93 boys	Flexible intramedullary nailing	12 months
Kirmani et al. ^(^ 12 ^)^	Prospective Study	N: 45 Femoral shaft fractures Sex: 16 girls and 29 boys	Flexible intramedullary nailing	12 months
Pogorelić et al. ^(^ 25 ^)^	Retrospective Study	N: 103 Dislocated femur fracture Sex: 27 girls and 76 boys	Flexible intramedullary nailing	92 months
Lu et al. ^(^ 26 ^)^	Retrospective Study	N: 28 Unstable fracture of the femoral shaft	Combined use of temporary external fixation and flexible intramedullary nailing	12 months
		Sex: 10 girls and 18 boys	Flexible intramedullary nailing	
Guo; Su ^(^ 14 ^)^	Retrospective Study	N: 165 Femoral shaft fractures Sex: 57 girls and 108 boys	Unilateral external fixation	19.7 months
Memeo et al. ^(^ 13 ^)^	Prospective Study	N: 62 Femoral shaft fractures Sex: 22 girls and 40 boys	Flexible intramedullary nailing	12 months
Li et al. ^(^ 27 ^)^	Retrospective comparative study	N: 15 Supracondylar fractures of the femur Sex: 9 girls and 6 boys	External fixation	24 months
Govindasamy et al. ^(^ 28 ^)^	Retrospective Study	N: 48 Femoral shaft fractures Sex: 18 girls and 30 boys	Flexible titanium intramedullary nailing	20 months
Rollo et al. ^(^ 29 ^)^	Retrospective Study	N: 38 Femoral shaft fractures Sex: 14 girls and 24 boys	Titanium flexible intramedullary nails and external fixators	14 months

### Evaluation of Interventions

Table 2 data show that the chosen studies obtained good and satisfactory results. Most authors reported no significant differences between complication rates across study groups. 

The most common reported complications referred to late or vicious union, superficial and deep infections, skin irritation, angular deformities, or discrepancy in length between lower limbs.

**Table 2.** Results of Interventions

Author/ Year	Result	Complications
Frumberg et al. ^22^	No significant differences in the rate of major complications or increases in angulation between the study and control groups.	One patient (16.7%). Occurrences: Increased anterior bowing of the femur.
Li et al. ^23^	Significant reduction in pain after surgery in both groups. The rate of major complications failed to significantly differ between the two groups.	15 patients (21.1%). Occurrences: Implant irritation; Surgical site infection; Vicious consolidation; Pseudoarthrosis or loss of reduction; Angular deformity.
Ulici et al. ^24^	21.0% of patients. Most patients were successfully treated by internal fixation with flexible nails.	29 patients (21%). Occurrences: Late consolidation; Axial deformities or discrepancies in lower extremity length.
Kirmani et al. ^12^	Results were excellent for 80% of patients.	12 patients (26.7%). Occurrences: Deep infection; Late consolidation; Superficial infection; Vicious consolidation;
	Fixation with a flexible intramedullary nail proved to be a safe method.	Limb length discrepancy; Skin irritation.
Pogorelić et al. ^25^	All patients achieved complete radiographic cure in an average of 8.5 weeks. After the removal of the nails, all patients regained full function of their limb, with no long-term consequences.	9 patients (8.49%). Occurrences: Skin irritations at the entry site; Valgus angulation; Implant protrusion; Refracture; Varus angulation; Delayed consolidation.
Lu et al. ^26^	All fractures healed, with no late union, malunion, or refracture. About 96.4% of the patients had excellent radiological results.	4 patients (14.3%). Occurrences: Pin-track infections; Temporary stiffness of the knee joint; 13-mm discrepancy in the lower limbs.
Guo; Su ^14^	About 14.5% of patients experienced refracture within one year of the removal of the external fixation.	24 patients (14.5%). Occurrence: Refracture.
Memeo et al. ^13^	All fractures healed within eight weeks after fixation, with no nonunion or delayed union. Children with transverse fractures had a shorter healing time.	24 patients (38.7%). Occurrences: Distal pain at the point of nail insertion; Superficial and deep infection; Knee stiffness; Loss of reduction; Proximal migration; Inflammatory reaction.
Li et al. ^27^	All fractures healed without delay in consolidation. No acute or serious complications were observed.	2 patients (13.3%). Occurrences: Superficial infection of the skin in the path of the nail.
Govindasamy et al. ^28^	All fractures healed radiologically with grade III callus formation from nine to 12 weeks. No late consolidation, nonunion, or refractures.	15 patients (31.3%). Occurrences: Limb shortening; Vicious consolidation; Infection of the protruding site of the nail; Nail migration; Skin irritation.
Rollo et al. ^29^	The end of follow-up found no significant rotational defects	14 patients (36.8%). Occurrences: Superficial infection in the access of the nails.
	Angulation or growth for both groups. For both groups, the range of hip and knee movement was superimposable. Flexible nailing showed a greater tolerability synthesis.	

### Results of the Meta-analysis

**Figure 1.**
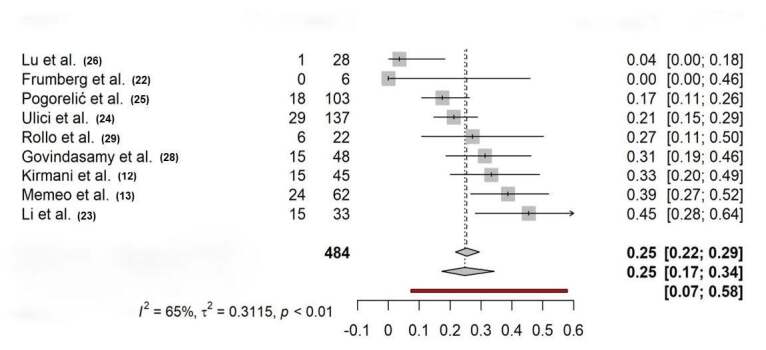
Forest plot showing the proportion of complications in studies that used flexible nailing without a control group.

 The first meta-analysis used individual proportions, combining the proportions or probabilities of an event occurring in several studies to calculate an overall proportion or probability. In total, nine studies used flexible nailing as treatment without a control group. The results of this meta-analysis ( Figure 1 ) indicated differences between the complications in each study (RR 0.25; CI-0.07; 0,58; p = 0.01). They found evidence of moderate heterogeneity across studies (I² = 65%, t² = 0.3115). 

 The second meta-analysis used individual proportions, combining the proportions or probabilities of an event occurring in multiple studies to calculate an overall proportion or probability. Overall, two studies used external fixation as treatment without a control group. The results of this meta-analysis ( Figure 2-A ) indicated no differences between complications in each study (RR 0.13; CI-0.09; 0,19; p = 1.00). Studies showed no evidence of heterogeneity (I² = 0%, t² = 0). 

**Figure 2-A:**
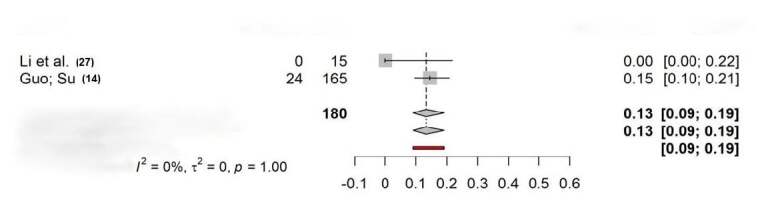

**Figure 2-B:**
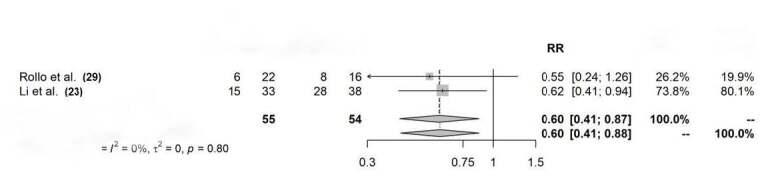
Forest plot showing the proportion of complications in studies that used external fixation without a control group (2-A) and studies that compared external fixation and flexible nailing in separate groups (3-B).

 The third meta-analysis used a meta-analysis of binary outcomes (occurrence or absence of complications in both groups: flexible nailing and external fixation). It considered both common and random effects to obtain its risk ratio and compare studies. It used two studies that compared external fixation and flexible nailing in separate groups. Results ( Figure 2-B ) indicated no differences between complications across studies (RR 0.60; CI-0.41; 0,87/0,88; p = 0.80). Studies showed no evidence of heterogeneity (I² = 0%, t² = 0). 

### Evaluation of the Quality of Studies

 After reading the 11 selected articles in full, this review evaluated their quality by the Newcastle-Ottawa scale ^12^ , attributing scores from 4 to 7 to each study ( Chart 1 ). Thus, the chosen studies ^14^^-^^24^ lie within the expected quality for this research. 

**Chart 1.** Assessment of bias according to the Newcastle-Ottawa Scale

Studies	Random sample	Unbiased sample	Sample with well-described subjects	Sample size	Unbiased evaluators	Response rate	Type of statistical test	Total
Frumberg et al. (22)	1	1	1	0	1	1	0	5
Li et al. (23)	1	1	1	0	1	1	0	5
Ulici et al. (24)	1	1	1	1	1	1	1	7
Kirmani et al. (12)	1	1	1	0	1	1	1	5
Pogorelić et al. (25)	1	1	1	1	1	1	1	7
Lu et al. (26)	0	1	1	0	1	1	0	4
Guo; Su (14)	0	1	1	1	1	1	1	6
Memeo et al. (13)	1	1	1	0	1	1	0	5
Li et al. (27)	0	1	1	0	1	1	0	4
Govindas amy et al. (28)	1	1	1	0	1	1	0	5
Rollo et al. (29)	0	1	1	0	1	1	0	4

Criteria to evaluate observational studies (maximum of 8 points): random sampling: 1 - yes, 0 - no

unbiased sampling: 1 - yes, 0 - no

sample with well-described subjects: 1 - yes, 0 - no

sample size: 1 - greater than or equal to 100 subjects, 0 - less than 100 subjects

PCAT utilization: 1 - PCATool standard version, 0 - PCATool adapted version

Unbiased evaluators: 1 - yes, 0 - no

Response rate: 1 - greater than or equal to 70%, 0 - less than 70%

Type of statistical test: 1 - T-test, 0 - other statistical tests

## DISCUSSION

 This systematic review aimed to compare the results of FSF treatment by ESIN and EF in children, highlighting associated complications among the outcomes. The main finding of this systematic review and meta-analysis refers to the statistically significant difference in the incidence of complications in flexible nailing ^12^^,^^13^^,^^22^^,^^23^^,^^24^^,^^25^^,^^26^^,^^28^^,^^29^ and the absence of statistical or clinical differences between the occurrence of complications for external fixation ^27^^,^^14^ or the comparison between interventions. ^23^^,^^29^

The literature shows that choosing the ideal treatment for these fractures remains controversial, constantly challenging the orthopedic community. The included studies generally classified their results as good and satisfactory. Most authors reported no significant differences between complication rates across study groups. The most common reported complications for both methods referred to delayed or vicious unions, superficial and deep infections, skin irritation, angular deformities, or length discrepancy between lower limbs.

 Chen et al.’s ^9^ meta-analysis reported ESIN outperforming EF in the early treatment of pediatric femoral fractures, comparing the discrepancy in observed length, hospital stay duration, time to clinical improvement, time to consolidation, and complication rates. Corroborating these findings, Kirmani et al. ^12^ reported that TEN nails offer a safe, reliable, and effective fixation method due to its simple application, lower degree of invasion, ease of implant insertion and removal, fast union, short rehabilitation, and less psychosocial stress for patients and their families. 

 Similarly, Ulici et al. ^24^ found that most patients (79%) were successfully treated by closed reduction and internal flexible nailing fixation, showing no complications. However, these researchers considered that two factors would be associated with complications: age above 11 years and/or weight above 50 kg. 

 Similarly, Frumberg et al. ^22^ reported that the use of TEN can offer an excellent surgical option to fixate fractures in pediatric patients weighing less than 45 kg with FSF and stable-length tibiae. They stress that patients’ weight should be carefully considered so gait forces fail to supplant the stability of the internal fixation provided by the intramedullary implant. Although ignored, this review believes that the diameter of the medullary canal and, therefore, the choice of the caliber of the intramedullary implants also configure determinant factors for obtaining adequately stable osteosynthesis. We stress that properly pre-shaping nails constitutes a fundamental step for the desired stability. 

Another available resource when choosing ESIN refers to End Cap Synthes, a cap that prevents TEN from sliding backward. Its rounded shape can adequately protect soft tissues. It should be used in unstable shaft fractures of the femur and tibia.

 According to Siddiqui et al. ^30^ , ESIN is the preferred implant to treat FSF in children. It uses three-point fixation, providing axial, translational, and rotational stability at the fracture site. It has minimal complication rates when applied properly. Pogorelić et al. ^25^ also emphasize that this device offers excellent functional and cosmetic results and enables early functional follow-up with rapid pain reduction. These authors concluded that, due to their excellent results, surgical stabilization of femoral fractures should be recommended for pediatric patients. However, we must report that removing these implants fails to always occur easily. The incision for removal can be much larger than the one used for insertion, and the adherence of bone to titanium often hinders removal. 

 Lu et al. ^26^ found that femoral fractures in children aged from five to 11 years can be treated by flexible nailing with temporary external fixation. It generally shows good or excellent functional and radiographic results and complication rates that resemble that of flexible nailing or external fixation alone. They observed only one case of discrepancy in lower limb length in patients treated with external fixation. However, the literature rarely reports the combined use of these devices. 

 Li et al. ^27^ also reported that external fixation has potential advantages: a minimally invasive approach, less blood loss, shorter operative time, and no need for secondary surgery for hardware removal. External fixation also produces satisfactory clinical results and can be comparable to flexible intramedullary nailing. 

 However, about 14.5% of the patients in Guo and Su ^14^ experienced a refracture within one year after EF removal. On the other hand, Li et al. ^27^ showed that external fixation techniques were considered the best option to treat deviated supracondylar femoral fractures in children. They found neither deformity, deep infections after surgery, nor symptoms requiring further treatment. The authors emphasize that the absence of infection should be attributed to the efficacy of prophylactic antibiotic therapy. Moreover, the external fixation group showed significantly lower amount of bleeding during the operation and the time of consolidation. 

 On the other hand, Li et al. ^23^ found that the irritation due to the implant was much greater in external fixation than in flexible intramedullary nailing due to the involvement of the thigh muscles that surround the distal femur. However, they emphasize that EF was routinely removed from seven to 12 postoperative weeks, whereas flexible nails were systematically removed from four to seven months. 

 On the other hand, Pogorelić et al. ^25^ found 8.49% (n: 107) of postoperative complications in patients who received flexible nailing: three skin irritations at the entry site, two valgus angulation cases, and one case of nail protrusion, refracture, varus angulation, and delayed union. All complications, except for refracture and valgus angulation, received conservative treatment, with no long-term consequences for patients after implant removal. 

 Moreover, Kirmani et al. ^12^ observed a case of deep infection in a patient with a type I open fracture, treating it with debridement and intravenous antibiotics without the need to remove the nail. They also emphasize that in open fractures with contamination, external fixation should be the preferred method of osteosynthesis to the detriment of flexible intramedullary nailing, potentially minimizing complications. 

 On the other hand, Ulici et al. ^24^ reported that unstable femoral fractures treated with flexible intramedullary nailing and immobilization with plaster casts show no higher risk of other complications, such as angular deformities, delayed consolidation, limb length discrepancies, or higher rates of premature ESIN removal. 

 Kirmani et al. ^12^ state that flexible intramedullary nailing should be avoided in patients weighing more than 45 kg and over 14 years of age as stability in these conditions follows failed weight bearing, leading to implant failure or malunion. Appropriate patient selection and strict adherence to basic techniques can decrease complication rates. Govindasamy et al. ^28^ also reinforce that the TEN nail offers efficacy in appropriately selected children. 

 Memeo et al. ^13^ also report that TEN nails configure an excellent internal fixation system if used by an experienced surgeon, showing very low complication rates. However, in older children weighing more than 50 kg, the authors advise the use of alternative techniques such as plate fixation or external fixation. 

 According to Govindasamy et al. ^28^ , the indication of ESIN continues to grow following reports of its advantages and low complication rates. They mention its immediate availability, caliber variability, and low cost as its main advantages. Complications are usually linked to improper techniques, which can be eliminated by strictly following the basic principles and technical aspects. 

 Corroborating these findings, Rollo et al. ^29^ show that ESIN and EF produce similar fracture consolidation and complication results. However, patients treated with a flexible nailing show a higher degree of satisfaction. Flexible nailing are currently considered the first choice for most pediatric femoral shaft fractures, offering many advantages and fewer complications. These findings agree with the results in our research. 

## CONCLUSION

The studies included in this systematic review found that flexible nailing and external fixation can provide good clinical and radiographic results in patients with pediatric femoral shaft fractures. However, the choice of treatment with flexible nailing receives greater acceptance than external fixation, and should be reserved for younger patients with lower age and weight, whereas treatment with external fixators remains the first choice in children aged over 11 years, weighing more than 50 kg, and showing multiple traumas or open fractures. We found that complications are usually associated with the inadequate application of the osteosynthesis technique, which can be solved by strictly following the basic principles of each technique. We stress that research with better scientific methodology, larger samples, and good-quality double-blind and randomized controlled designs can compare and confirm the efficacy of these techniques.
